# Endovascular treatment of chronic mesenteric ischemia in an adolescent male: case report

**DOI:** 10.12688/f1000research.2-277.v1

**Published:** 2013-12-17

**Authors:** Sadiq Muhammed Al-Hammash, Abd El-Salam Dawood Al-Ethawi, Kasim Abbas Ismail

**Affiliations:** 1 Ibn Al-Bittar Center for Cardiac Surgery, College of Medicine, Al-Mustansiriyah University, Baghdad, Iraq

## Abstract

Chronic mesenteric ischemia (CMI) is a condition characterized by inadequate blood flow to the bowel resulting from stenosis of one or more of the three mesenteric arteries. Fibromuscular dysplasia is the most common cause in children and young adults; whereas atherosclerosis is the most common cause in the elderly. Treatment is mandatory in symptomatic patients, because it may lead to malnutrition, bowel infarction or even death.Herein, we present the case of a 14-year old male, diagnosed with CMI who underwent percutaneous balloon angioplasty (PTA) followed by stent placement with immediate positive results.

## Introduction

Chronic mesenteric ischemia (CMI) is a condition characterized by inadequate blood flow to the bowel resulting from stenosis of one or more of the three mesenteric arteries: the celiac artery (CA), the superior mesenteric artery (SMA), and the inferior mesenteric artery (IMA). Many case-series include patients with ischemic bowel due to stenosis of a single artery ^1– 3^.

Fibromuscular dysplasia (FMD) is a rare disorder of medium-sized arteries with no signs of inflammation or atherosclerosis ^4^. It is the most common cause of stenosis in children and young adults. The reported worldwide incidence is around 1% ^5^.

Renal arteries (60–75%) and cerebral arteries (25–30%) are the most commonly involved vasculatures; followed by the mesenteric, iliac, and subclavian arteries ^5^.

In those older than 60 years atherosclerotic plaques are the most common cause of stenosis of the mesenteric arteries, with females affected three times more often than males ^6^.

Symptomatic untreated bowel ischemia may cause malnutrition or acute bowel ischemia with infarction –a complication that is associated with a bad prognosis ^6^. A fatal outcome has been reported in some cases ^7^. Therefore, treatment is mandatory in symptomatic cases.

The gold standard therapeutic option is surgical (either a bypass or endarterectomy) with a combined morbidity of 15–47% and a mortality of 0–17% ^8– 12^. Stenoses of the mesenteric vessels are focal, and found at the ostium or proximal part of the artery – two features that make them accessible to transluminal therapy. Endovascular treatment of bowel ischemia was first described in 1980 ^13^ and subsequently good results were found in two small studies ^14, 15^. Encouraged by these results, several centers, including ours, started using transluminal angioplastic techniques as the first line therapy for mesenteric ischemia.

Herein, we present the case of a 14-year old Arabic Muslim male from the center of Iraq who was diagnosed with chronic mesenteric ischemia and who underwent a percutaneous balloon angioplasty (PTA) followed by stent placement with immediate positive results.

## Case report

The condition started in infancy when the child had recurrent attacks of respiratory tract infections and exhibited a failure to thrive. He was diagnosed at that time as a case of moderate-sized patent ductus arteriosus (PDA) and small restrictive perimembranous ventricular septal defect (VSD). The patient had been prescribed anti- heart failure medications (in the form of digoxin oral drops (10 mcg/kg/day in two divided doses, furosemide 1 mg/kg bid by mouth (PO), and captopril 0.5 mg/kg by mouth per day (QD PO)) and the patient had frequent follow-up visits. His PDA had been closed via a transcatheter approach using an Amplatzer ^®^ ductal occluder device at the age of 8 months and the VSD closed spontaneously as suggested by clinical follow-up exams and confirmed by echocardiography. The child’s medication was stopped at the age of 2 years and he was thriving well.

One year ago (at the age of 13), the patient started to develop post-prandial nausea and abdominal pain necessitating a complete work-up including investigations to exclude peptic ulcer disease (PUD) as a cause of these symptoms.

The patient was referred back to our center by his pediatrician to exclude any complicating event associated with his ex-PDA and VSD. An initial clinical examination was unremarkable. A screening echocardiography was performed that showed turbulence in the abdominal aorta near the origin of the celiac artery (CA), which raised the possibility of chronic mesenteric ischemia as the cause of his symptoms.

**Figure 1.**
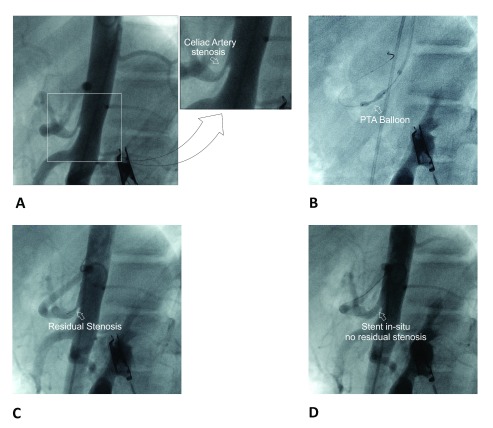
Percutaneous balloon angioplasty with stent placement for celiac artery stenosis. **A**. Non-selective angiography (lateral view) showing ostial and proximal segment stenosis of the celiac artery. (The inset shows a close-up view of the stenosis; arrow). **B**. PTA balloon inflated showing “waist” formation (arrow). **C**. Residual stenosis (arrow) after initial PTA. **D**. Stent *in-situ* with no residual stenosis (arrow).

Catheterization was done and a 6 F short sheath inserted in the right femoral artery. A non-selective abdominal aortography followed by selective angiography using a 6 F Judkins Right ^®^ (JR) catheter confirmed the presence of a narrowing at the ostial and proximal segments of the celiac artery. The length of the narrowed segment, the diameter of the stenosed segment and the nearby normal segment were measured, expert consultation was done, and the patient was scheduled for subsequent therapeutic catheterization including percutaneous balloon angioplasty (PTA) with or without stent placement ( Figure 1A).

Six weeks later (after being prepared with aspirin 100 mg QD PO for seven days and clopidogrel 75 mg QD PO for three days, a 6 F short sheath introducer was inserted in the right femoral artery and another 6 F hydrophilic short sheath introducer was inserted in the right radial artery after confirming palmar arch patency using modified Allen's test ^16^ and skin infiltration with 1.5 ml lidocaine as a local anaesthetic. Heparin 5000 IU was given intravenously immediately after inserting the radial sheath and 200 mcg nitroglycerine was administered via the sheath. The femoral sheath was used for monitoring, and non-selective angiography (for proper localization of the balloon), while the radial sheath was used to deliver the balloon (with or without the stent) into the celiac artery. A 6 French guiding catheter was passed over a 0.030" exchange guide wire (GW) reaching up to the ostium of the CA from the radial artery. It was then replaced with a 0.014" soft-tipped GW, which was passed a few centimeters within the CA. A 4 mm diameter percutaneous coronary angioplasty (PTCA) balloon was passed over the 0.014' GW and dilatation of the ostial and proximal segment of the CA was done (reaching nominal pressure and kept inflated for 30 seconds) ( Figure 1B and C). A residual stenosis of about 40% was noticed after the balloon angioplasty, necessitating the use of a Promus ^®^ drug-eluting stent (PROMUS Element™ Plus; *Everolimus-Eluting Platinum Chromium Coronary Stent System*), premounted on a balloon, measuring 4 mm in diameter and 12 mm in length. The balloon was inflated just-below burst pressure and kept inflated for 30 seconds. An angiography conducted after this procedure, showed a diameter equal to the nearby normal segment ( Figure 1D).

The radial artery sheath was removed immediately after completing the procedure and hemostasis was secured using a TR band ^®^ (compression strap) and a femoral artery sheath, removed after 60 minutes.

There were no immediate post-operative complications. The patient was discharged on the second day after the operation and was instructed to take both aspirin 100 mg/day QD PO and clopidogrel 75 mg/day QD PO for the following six months followed by use of aspirin alone for life. In the subsequent follow-up visits conducted one- and six-months after the procedure, there was neither post-prandial abdominal pain, nor nausea and the patient gained 7 kilograms weight over 6 months.

## Discussion

Untreated chronic mesenteric ischemia (CMI) can lead to symptoms of post-prandial abdominal pain, malnutrition, or even death. Therefore, a symptomatic patient needs treatment. The conventional treatment is surgical via a bypass surgery with or without endarterectomy ^6^. Transluminal endovascular intervention, first described in 1980 ^13^ and the good results obtained by two small studies encouraged many centers- including ours- to adopt the endovascular treatment as the first line therapy.

The aims of therapy are to: 1) achieve symptomatic resolution, 2) prevent bowel infarction and resultant morbidity and mortality and 3) prevent and treat malnutrition. Some groups also recommend prophylactic revascularization even in asymptomatic patients who are due for an operation in the aorta (e.g. aneurysm, coarctation, etc.) or before being scheduled for major abdominal surgery that might jeopardize the collateral circulation ^6^.

The patient had a moderate–sized PDA and small restrictive VSD diagnosed in infancy. The former was closed by a transcatheter approach and the latter closed spontaneously. Up to the time of writing this article, the authors were unable to find a study showing an association between these two defects and mesenteric ischemia due to celiac artery stenosis. As such these findings appear coincidental.

Two catheterization sessions were needed in our patient because expert consultation and support was needed. However, if the patient is well-prepared in terms of antiplatelet medications (seven days of aspirin and three days of clopidogrel in our center); equipment and expert consultation is available, one catheterization session might suffice for confirming the diagnosis, planning, measuring and proceeding with angioplasty with or without stent placement.

Since the initial PTA result was unsatisfactory with 40% residual stenosis, a drug-eluting covered stent (PROMUS Element™ Plus; *Everolimus-Eluting Platinum Chromium Coronary Stent System)* with a length ensuring 1 mm protrusion in the aortic lumen and 1–2 mm coverage of the nearby normal-width segment was inserted. The diameter chosen depends on the diameter of the nearby normal segment (we added 1 mm to the diameter of the normal segment).

Some centers also recommend using stents in the following situations: 1) lesions located at the ostium or eccentric lesions, 2) History of dissection after a previous angioplasty and 3) Chronic stenosis ^17^.

Since the take-off of the celiac artery has an acute angle to the aortic wall, the superior approach (i.e. radial) provides a smoother curve and easier access of this artery. For that reason we chose the right radial artery after using a modified Allen test ^16^ to confirm patency of the palmar arch.

The use of vasodilators (nitroglycerine) given in the sheath is necessary to prevent radial artery vasospasm.

## Conclusion

Symptomatic CIM can be found in the pediatric age group. We contribute this case report to the growing body of information regarding the role and effectiveness of endovascular treatment of this condition. Further research is needed to elucidate the “long-term” efficacy and safety of this therapeutic option.

## Consent

Written informed consent for publication of clinical details was obtained from the patient's parents.
