# Supplementary material for: Endovascular treatment of chronic mesenteric ischemia in an adolescent male: case report
Source: F1000Res. 2013 Dec 17;2:277. [Version 1] doi: 10.12688/f1000research.2-277.v1 (PMC4103498; doi:10.12688/f1000research.2-277.v1)
Supplement: Peer review history

# Peer review: Referee response for version 1

**DOI:** 10.5256/f1000research.3190.r4650

**Published:** 2014-05-13

**Authors:** Lefeng Qu

**Affiliations:** 1 Department of Vascular Surgery, Shanghai Hospital, Second Military Medical University, Shanghai, China

This article reported a case of a 14-year old male, diagnosed with CMI who underwent PTA followed by stent placement with immediate positive results.

The history was reported clearly, and the procedure was described in detail.

The authors also pointed out their experiences concerning the tips of the procedure, such as choosing the right radial artery as the approach to overcome the acute angle of the celiac artery, and the use of nitroglycerine to prevent radial artery vasospasm.

Generally, this is an interesting article, and has certain merits for indexing.

I have read this submission. I believe that I have an appropriate level of expertise to confirm that it is of an acceptable scientific standard.

# Peer review: Referee response for version 1

**DOI:** 10.5256/f1000research.3190.r4553

**Published:** 2014-04-25

**Authors:** Madathipat Unnikrishnan

**Affiliations:** 1 Department of Cardiovascular and Thoracic Surgery, Sree Chitra Thirunal Institute of Medical Sciences and Technology, Trivandrum, India

The authors present an excellent and well-written case  of a 14 year old boy who reported with features of mesenteric angina due to ostioproximal stenosis of the coeliac trunk managed by percutaneous angioplasty combined with stenting. Technical success was followed by clinical relief of symptoms along with considerable weight gain for the boy as expected.

However, I wish to place a few of my thoughts and suggestions regarding this case scenario:

 

- The last line of the introduction (just prior to Case report) is redundant.
- Isolated coeliac artery stenosis is rarely the cause for mesenteric angina in the presence of a full calibre superior mesenteric artery. Also ostioproximal lesion is not typical of FMD which usually involves the body of an artery (Renal /internal carotid).
- In this 14 year old boy, having had congenital cardiac (VSD and PDA)lesions, rarely found coeliac artery syndrome might have been the cause that had given rise to the symptoms described.

I congratulate the authors and suggest they also discuss the possibility or otherwise of coeliac artery syndrome in discussion which would make this a tidy and valuable contribution.

I have read this submission. I believe that I have an appropriate level of expertise to confirm that it is of an acceptable scientific standard.

# Response

**Published:** 2014-04-28

**Authors:** Madathipat Unnikrishnan

**Affiliations:** Sree Chitra Thirunal Institute of Medical Sciences and Technology, India

In continuation to my referee report, in the Conclusion the authors should change *'CIM'* to ' *CMI'*
